# Increased Rate of Arterial Stiffening with Obesity in Adolescents: A Five-Year Follow-Up Study

**DOI:** 10.1371/journal.pone.0057454

**Published:** 2013-02-22

**Authors:** Frida Dangardt, Yun Chen, Krister Berggren, Walter Osika, Peter Friberg

**Affiliations:** Department of Molecular and Clinical Medicine, Clinical Physiology, Sahlgrenska Academy at the University of Gothenburg, Gothenburg, Sweden; Medical University Innsbruck, Austria

## Abstract

**Background:**

We prospectively and longitudinally determined the effects of childhood obesity on arterial stiffening and vascular wall changes. Changes in arterial stiffness measured as pulse wave velocity (PWV) and vascular morphology of the radial (RA) and dorsal pedal arteries (DPA) were examined in obese adolescents compared to lean subjects in a 5-year follow-up study.

**Methodology/Principal Findings:**

A total of 28 obese subjects and 14 lean controls participated in both baseline (14 years old) and follow-up studies. PWV was measured by tonometer (SphygmoCor®) and recorded at RA and carotid artery, respectively. Intima thickness (IT), intima-media thickness (IMT) and RA and DPA diameters were measured using high-resolution ultrasound (Vevo 770™). Over the course of 5 years, PWV increased by 25% in the obese subjects as compared to 3% in the controls (p = 0.01). Diastolic blood pressure (DBP) increased by 23% in the obese subjects as opposed to 6% in controls (p = 0.009). BMI increased similarly in both groups, as did the IT and IMT. The change in PWV was strongly associated to the baseline BMI z -score (r = 0.51, p<0.001), as was the change in DBP (r = 0.50, p = 0.001).

**Conclusions/Significance:**

During the transition from early to late adolescence, there was a general increase in arterial stiffness, which was aggravated by childhood obesity. The increase in arterial stiffness and DBP after 5 years was closely correlated to the baseline BMI z -score, indicating that childhood obesity has an adverse impact on vascular adaptation.

## Introduction

The paediatric origin of atherosclerosis is well accepted. Studies of cardiovascular risk factors that are initiated in childhood provide evidence that childhood risk exposure predicts subclinical markers of atherosclerosis in adulthood [1], [2], [3].

There is a known association between childhood body mass index (BMI) and the risk of having a coronary heart disease event (nonfatal or fatal) in adulthood [4]. Hence, childhood obesity confers an increased risk of vascular change and adult cardiovascular disease. We have previously shown that obese children have increased left ventricular mass [5]. This mass increase might be an early consequence of the prevalence of increased blood volume and hyperkinetic circulation in the obese state, in which the excessive fat mass constitutes an ‘extra organ’, thus amplifying the demand for an augmented cardiac output to provide an adequate blood flow throughout the body [6].

In adults, vascular health assessment such as arterial stiffness, measured as pulse wave velocity (PWV), have been shown to be strong predictors of future cardiovascular events [7], [8] PWV is positively associated with BMI in adulthood, and a reduction in obesity from childhood to adulthood is associated with lower PWV in adulthood [9].

Radial artery intima thickness may indicate early atherogenic processes in the vascular wall of obese children and adolescents. By using a new ultrasound system with very high resolution and a discriminatory power down to 30 µm [10], we were able to show that obese children have increased arterial intimal thickness when compared to lean controls, a change that could not be detected by the traditional measurements of the intima-media complex [11].

Adolescence, especially between the ages of 13 and 18, brings an onset of hormonal changes and growth that are likely to considerably affect the vasculature. The changes in insulin and growth hormone at least moderates changes in blood pressure [12], whereas other cardiovascular changes have been poorly examined. To study how and to what extent these effects of adolescent transition, in combination with obesity, influence the peripheral and central vasculature, we undertook a 5-year follow-up study of obese adolescents and compared them to lean subjects.

We hypothesised that obese adolescents, as compared to lean controls, would have larger vessel diameters, increased intimal thickness, and increased arterial stiffness as early signs of altered vascular morphology and function. Hence, we examined the 5-year changes in vascular morphology of the radial (RA) and dorsal pedal (DPA) arteries and arterial stiffness, measured as PWV, in obese adolescents and lean controls.

## Material and Methods

### Study design

Obese/overweight adolescents and age- and sex-matched lean controls were examined at baseline and after 5 years. During this time they did not participate in any organised intervention.

### Subjects

At baseline, subjects who were referred to the Queen Silvia Children's Hospital in Gothenburg, Sweden for obesity treatment were enrolled to participate in the study. Age- and sex-matched controls were recruited from schools within the Gothenburg area. Obesity was defined by the International Obesity Task Force (IOTF) criteria [13], and the BMI z-score was calculated with a Swedish population as a reference [14]. Of the initial 51 participants, 42 agreed to participate in the 5-year follow-up study. Of the 9 dropouts (6 obese), 5 were due to difficulty in locating a current address, 2 did not want to participate, and 1 lived abroad. Of the 42 follow-up participants, 24 were classified as obese (BMI z-score >2.5), 4 as overweight (BMI z-score 1.5–2.5) and 14 as lean (BMI z-score <1.5) at baseline. The overweight and obese were consolidated into one group referred to as ‘obese’. Informed consent and written protocols, approved by the ethics committee at Sahlgrenska Academy in Gothenburg, were presented to the adolescents and their parents. Written consent was obtained from both the adolescents and their parents when applicable. The trial was conducted in accordance with Good Clinical Practice.

### Study protocol

At the baseline and the 5-year follow-up, all participants underwent a physical examination. Height and weight were measured with a stadiometer and an electronic scale. Resting blood pressure levels were measured from the right arm using an electronic sphygmomanometer (Welch Allyn Inc., NY, USA). After 15 min of rest in a supine position, three separate readings were taken 2 min apart, and the average of the second and third readings was used for analysis.

Measurements of PWV were performed using a pressure tonometer to transcutaneously record the pressure pulse waveform in the underlying artery (SphygmoCor® system, AtCor Medical, Australia), as previously described [11]. In short, records were made simultaneously with an ECG signal, which provided an *R*-timing reference [15]. Pulse wave recordings were performed consecutively at two superficial artery sites (carotid-radial segment). Integral software was used to process each set of pulse wave and ECG data to calculate the mean time difference between *R*-wave and pulse wave on a beat-to-beat basis, with an average of 10 consecutive cardiac cycles. The PWV was calculated using the distance and mean time difference between the two recorded points. Quality indices, included in the software, were set to ensure the uniformity of data. In an earlier study, we investigated the reproducibility of the radial-carotid PWV measurements in a separate group of healthy children and adolescents (n = 10). The same examiner studied the subjects on two occasions separated by a 6-month interval. The coefficient of variation was 9% [11].

The ultrasound measurements were performed using high-resolution ultrasound of 55 MHz (Vevo 770™ Visualsonics Inc., Toronto, Canada), validated for use in human peripheral arteries [10]. Right side RA and DPA of resting subjects in a supine position were scanned to obtain 2-D images, which were subsequently analysed off-line. RA was investigated 1–2 cm proximal to the skin fold separating the palma manus from the regio antebrachii anterior, and DPA was measured above the proximal first metatarsal bone in the foot. At the thickest part of the far wall, judged visually, four consecutive beats were recorded in real time and saved [10].

Intimal thickness (IT) was defined as the total thickness measured with callipers within a higher resolution zoom. Three measurements of the IT were performed in systole at the artery's largest diameter, judged visually. IMT was defined as the distance from the leading edge of the luminal-intimal interface to the leading edge of the medial-adventitial interface. Lumen diameter was defined as the distance between the leading edges of the intimal-luminal interface of the near wall and the luminal-intimal interface of the far wall [16]. From the in vitro validation experiments presented in our previous work, we revealed a systematic error of overestimation of the IT by 18 µm.

### Statistical analysis

Statistical analyses were performed with the IBM^®^ SPSS^®^ Statistics 19.0 (SPSS Inc.). As the study consists of few subjects and many of the variables are not normally distributed, non-parametric tests were used for statistical analysis. For related samples, the Wilcoxon signed ranks test was used to compare baseline and follow-up data, and for independent samples, the median test was used to compare between groups. The relationship between two variables was assessed from bivariate scatter plots and from the calculation of Pearson's correlation coefficient. All results are expressed as median and range. A p<0.05 was considered statistically significant.

## Results

### Anthropometry (Table 1)

**Table 1:** Anthropometry and vascular measurements at baseline and five-year follow-up in obese/overweight and lean controls.

		Obese/overweight (n = 28, 17 Female, 11 Male)	Lean controls n = 14, 9 Female, 5 Male)	p-value
Age (years)	Baseline	13.8 (10.2–17.6)	13.8 (11.5–16.1)	0.99
	Follow-up	18.5 (15.3–23.1) ‡	19.0 (16.7–21.4) ‡	
Height (m)	Baseline	1.63 (1.38–1.88)	1.65 (1.49–1.85)	0.51
	Follow-up	1.71 (1.55–1.90) ‡	1.71 (1.62–1.94) †	
Weight (kg)	Baseline	78.0 (40.5–119.0)	54.5 (42.0–69.0)	1.00
	Follow-up	95.0 (69.8–168.5) ‡	67.3 (50.5–94.4) ‡	
BMI (kg/m^2^)	Baseline	29.6 (21.3–39.9)	19.8 (17.4–22.4)	0.19
	Follow-up	34.5 (24.2–51.6) ‡	22.4 (18.5–23.0) ‡	
SBP (mmHg)	Baseline	115 (93–136)	111 (90–122)	0.28
	Follow-up	122 (100–155) ‡	116 (97–134) *	
DBP (mmHg)	Baseline	60 (42–72)	62 (47–78)	0.009
	Follow-up	74 (55–85) ‡	67 (57–72)	
Intima of radial artery (mm)	Baseline	0.05 (0.05–0.06)	0.05 (0.03–0.06)	0.23
	Follow-up	0.06 (0.05–0.14) ‡	0.08 (0.05–0.09) ‡	
IMT of radial artery (mm)	Baseline	0.20 (0.14–0.28)	0.22 (0.14–0.28)	0.04
	Follow-up	0.22 (0.16–0.30)	0.20 (0.17–0.27)	
Diameter of radial artery (mm)	Baseline	1.7 (1.0–2.6)	1.7 (1.0–2.2)	0.65
	Follow-up	2.1 (1.6–2.7) ‡	1.9 (1.6–2.8) ‡	
Intima of dorsal pedal artery (µm)	Baseline	0.06 (0.03–0.07)	0.06 (0.03–0.06)	0.85
	Follow-up	0.08 (0.05–0.09) ‡	0.08 (0.06–0.09) ‡	
IMT of dorsal pedal artery (mm)	Baseline	0.23 (0.16–0.31)	0.20 (0.12–0.29)	0.12
	Follow-up	0.22 (0.14–0.34)	0.23 (0.20–0.30)	
Diameter of dorsal pedal artery (mm)	Baseline	1.4 (0.4–2.2)	1.0 (0.7–2.1)	0.90
	Follow-up	1.5 (0.5–2.7)	1.5 (0.7–1.9) *	
Pulse wave velocity (m/s)	Baseline	6.5 (4.6–8.3)	6.6 (5.2–7.5	0.01
	Follow-up	8.1 (5.7–11.3) ‡	6.8 (6.1–9.1) *	

* = p<0.05, † = p<0.01, ‡ = p<0.001 represents differences between baseline and follow-up within groups, p-values presented as numbers in the last column represents difference in change from baseline to follow-up between groups.

Wilcoxon signed ranks test was used for related samples, Independent samples median test was used for between – groups comparison. Values are presented as median (min–max). BMI: body mass index, SBP: systolic blood pressure, DBP: diastolic blood pressure, IMT: intima media thickness.

The increase in height, weight and, consequently, BMI over the 5 year follow- up period was similar in lean and obese/overweight subjects. Of 13 subjects who were lean at baseline, one became overweight and one became obese at follow-up. Of 29 subjects who were obese/overweight at baseline, one became lean at follow-up. We chose to categorise subjects as lean control or obese/overweight according to the baseline BMI z -score because we were interested in the impact of childhood obesity on vascular health.

### Vascular measurements (Table 1)

**Figure 1:**
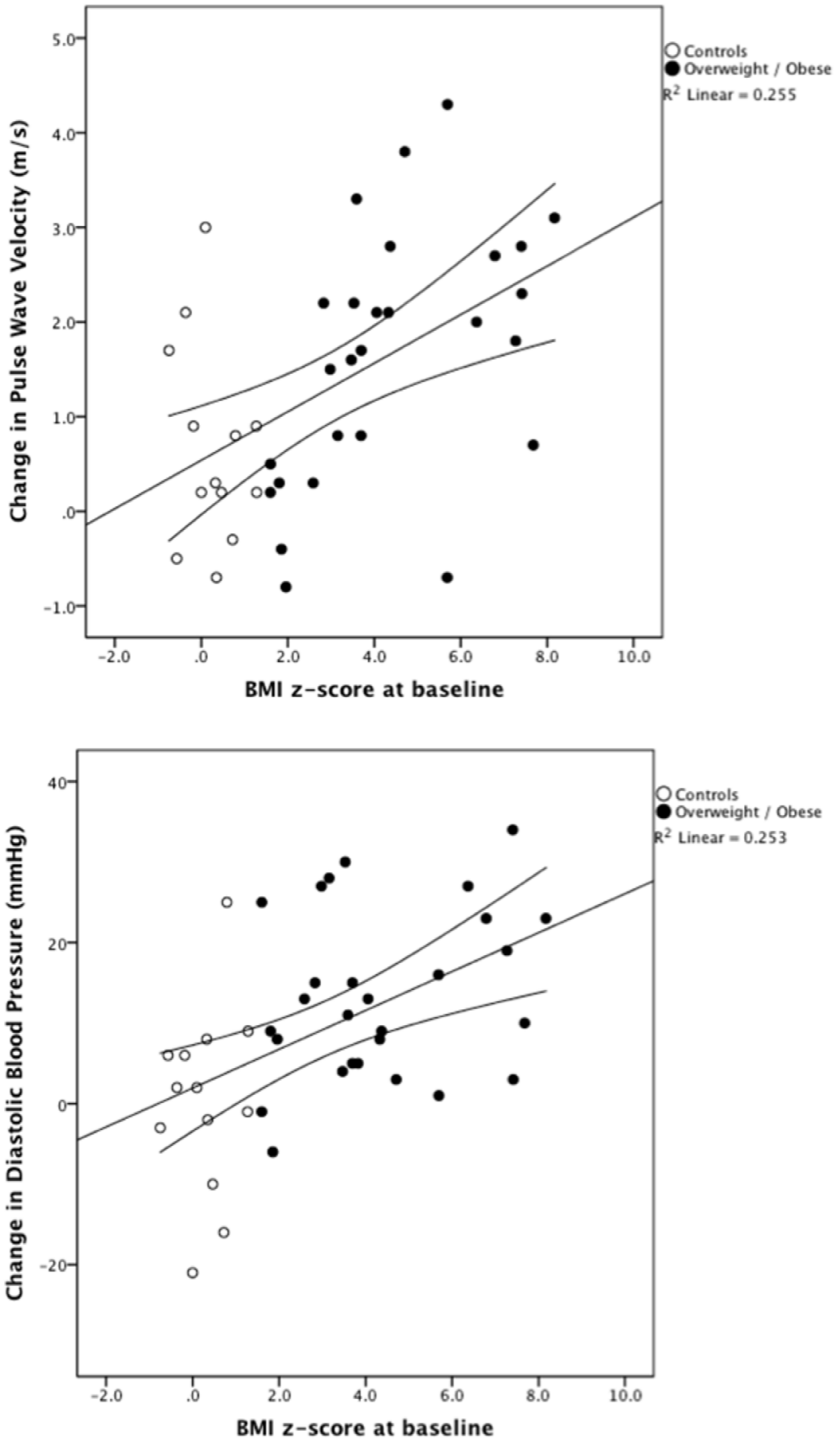
Association between body mass index z score at baseline and five-year change in pulse wave velocity (upper panel) and diastolic blood pressure (lower panel).

At baseline, there was no statistically significant difference in systolic blood pressure (SBP), diastolic blood pressure (DBP) and PWV. DBP increased by 23% in the obese and by 8% in the controls (Δ change p = 0.001 and 0.04 for obese and controls, respectively) over 5 years. SBP increased significantly to a similar extent in both groups: by 6% in the obese and 5% in the controls. PWV increased by 25% in the obese as compared to 3% in the controls (Δ change p<0.0001 and 0.04 for obese and controls, respectively). The change in PWV was strongly associated to baseline BMI z -score (r = 0.51, p<0.001, Figure 1, upper panel), as was the change in DBP (r = 0.50, p = 0.001, Figure 1, lower panel). The change in PWV was independent of gender and weight gain.

The RA diameter, which was slightly larger in the obese than in the controls at baseline, increased significantly in both obese and controls; however, it did not change significantly between groups. The diameter of DPA, also moderately larger in the obese than in controls at baseline, increased significantly only in the controls, but no significant difference in change could be found between groups. The change in PWV was negatively associated to the change in RA diameter (r = −0.33, p = 0.05), and this change was positively associated with the change in DBP, although not significant (r = 0.30, p = 0.06). The IT of both RA and DPA increased to a similar extent in both obese subjects and controls. The IMT of the RA slightly increased in obese subjects, and the change was significantly different between obese subjects and controls (p = 0.04). No difference in change regarding DPA IMT could be found between groups.

## Discussion

This study is, to the best of our knowledge, the first study to show the development of both vascular function and morphology during adolescence in obese subjects as compared to age- and sex-matched lean controls. We found that obese adolescents had a substantial increase in PWV as a measure of arterial stiffness, as early as 14 to 19 years of age, beyond what was observed in the lean controls, independent of weight gain during the follow -up period. Previous cross-sectional studies have found that childhood obesity is associated with high arterial stiffness, high IMT in peripheral and carotid arteries, and low endothelial function already by late teenage years [17], [18], [19]. This finding suggests that the degree and duration of obesity are important factors for determining the cardiovascular phenotype, especially during the important transition from child- to adulthood, where hormonal changes and growth are influential. Our results from this longitudinal study provide further support to these studies by showing that both DBP and PWV, as markers for arterial stiffness, increase significantly more in the obese subjects than in the lean controls. Thus, during transition from early to late adolescence, obesity aggravates arterial stiffening.

In addition to the increase in PWV, the obese adolescents show a 22% increase in DBP over the 5-year period, while no change occurred in the lean adolescents. Apparently the changes in PWV and DBP are closely related to, and also dependent on, the diameter of the blood vessels, which secondarily have to accommodate the physiological increase of blood volume. The obese subjects had larger peripheral arterial vessel diameter than the lean controls at baseline. This finding might indicate that obese adolescents' vascular systems initially adapt to accommodate the larger blood volume, generated by the marked increase in fat mass, by overall ‘vasodilatation’ and increased Windkessel function, as previously described when the baseline investigation of this study was presented [11]. Interestingly, in recently published material from the ALSPAC study, Donald and co-workers supported our previous findings by their observation of a negative association between BMI and PWV in a large cohort of pre-pubertal children [20], which strengthen the theory of a ‘chronic vasodilatation’ in the early obese phase. However, it seems that there is a limit to this physiological adaptive response to obesity, such that when the limit is reached during adolescence, diastolic blood pressure increases, and consequently, the previously augmented Windkessel effect is lost, evidenced here by the observed increase of arterial stiffness.

Arterial stiffness, as reflected by our measurements of the PWV, is determined by the arterial wall structure in relation to the arterial blood pressure and has been shown to be a strong predictor of cardiovascular mortality in hypertensive adults [21]. Studies have used various methods and arterial sites to assess arterial stiffness in obese children and adolescents. Tounian et al. found increased carotid artery stiffness in obese children by using ultrasound parameters to calculate vessel wall distensibility [22].This method might present a problem because the obese children show signs of early adaptive vasodilation and, therefore, have an increased baseline diameter. This leaves less capacity for distensibility even if the vessel is not stiff per se. Another problem is that the method is very user-dependent and has relatively low resolution, which causes less accurate measurements. The ALSPAC study, as mentioned above, supports the theory on adaptive vasodilation by showing a strong correlation between PWV and brachial diameter [20]. We found that there was a larger increase over time in PWV and diastolic blood pressure in obese adolescents than the lean controls, indicating that childhood obesity has an impact on arterial stiffness. The altered metabolic and/or haemodynamic demands, such as increased overall vasodilatation, blood volume, and cardiac output, which together with decreased total peripheral resistance have been documented in adult obesity [6]. In other words, as our baseline data suggest, arterial vasodilatation may be a physiological consequence of the hyperinsulinemic state and not a pathological process per se, and it might be reversible should the individual become less obese from childhood to adulthood, as shown in the recent study by Raitakari and co-workers [9]. In the long term, a condition with dilated vessels in which the structural component does not match the increased vessel diameter may lead to augmented wall tension, as suggested by the increased PWV in the obese subjects in this study. This in turn could result in impairment of vascular features and dysfunctional regulation, such as hampering endothelial responses and, consequently, arterial stiffening and possibly hypertension. The inflammation prevalent in the obese children, which increases in adolescence, also contributes to the structural adaptation of the vessels preceding hypertension, as described by Folkow and others [18], [23].

## Conclusion

Compared to the lean controls, the obese subjects presented rapidly increasing arterial stiffness over a 5-year period from early to late adolescence, indicating that this is an important transitional phase in which vascular changes occur. The increase in arterial stiffness and DBP after 5 years correlates closely to the baseline BMI z -score, indicating that childhood obesity has an adverse effect on vascular adaptation and that baseline BMI z -score predicts PWV.

### Perspectives

Our results suggest that important vascular changes in obese individuals occur as early as in adolescence. Further studies are needed to see if these changes are modifiable by weight loss or other treatments.
